# Case Report: A rare case of cervical lymph node metastasis from prostate cancer suspicious for combined lung cancer

**DOI:** 10.3389/fonc.2025.1615256

**Published:** 2025-10-13

**Authors:** Fei-Yan Zhou, Yue Du, Fang-Hua Song, Jing-Yang Sun

**Affiliations:** ^1^ Department of Oncology, Xinhua Hospital Affiliated to Dalian University, Dalian, Liaoning, China; ^2^ Department of Pathology, Xinhua Hospital Affiliated to Dalian University, Dalian, Liaoning, China

**Keywords:** prostate cancer, cervical lymph node metastasis, lung cancer, multiple primary malignancies, case report

## Abstract

Cervical lymph node metastasis as the initial manifestation of prostate cancer is uncommon, and the diagnostic and therapeutic processes become considerably more complex when primary lung malignancy is also suspected. Immunohistochemical (IHC) techniques are critical in the diagnosis of multiple primary malignancies, in particular when histopathologic access is limited. We report on a 74-year-old male patient who presented with respiratory symptoms. Computed tomography revealed a 6.4-cm × 4.7-cm mass in the upper lobe of the right lung with cervical lymph node metastasis. The initial diagnosis of metastatic adenocarcinoma was established by cervical lymph node biopsy. During follow-up, the patient developed progressive dysuria, and prostate cancer was ultimately confirmed by prostate biopsy. IHC analysis of the cervical lymph node specimen revealed the following profile: prostate-specific antigen (PSA) (focally positive, 3+), P504S (diffusely positive, 3+), TTF1 (−), CK7 (−), CK20 (−), napsin A (−), and P40 (−). This IHC profile definitively established the prostatic origin of the metastatic carcinoma in the cervical lymph node. This case highlights the value of employing therapeutic diagnostic strategies when definitive histopathologic access is challenging and provides insights into the management of atypical metastatic prostate cancer and multiple primary cancers.

## Introduction

Prostate cancer (PCa) is the most prevalent urological malignancy in men, and its global incidence is expected to increase to approximately 1.7 million new cases by 2030, with its development closely associated with age, genetic predisposition, and abnormalities in the androgen signaling pathway (1, 2). The bone is the preferred site of metastasis in PCa, with 80% of patients with advanced PCa developing bone metastasis (3). Lymph node metastasis is predominantly pelvic lymph node metastasis (4), whereas the incidence of neck lymph node metastasis is less than 1%, which is often suggestive of the aggressive biological behavior of the tumor. This atypical metastatic mechanism may be related to a retrograde spread due to obstruction of cervical lymphatic drainage, which is easily confused with metastasis from primary tumors in the head and neck region in clinical practice, leading to delayed diagnosis (5). When patients with PCa are complicated by lung-occupying lesions, the differential diagnosis faces a double challenge: on the one hand, it is necessary to differentiate between the primary lung cancer and prostate metastases; on the other hand, it is necessary to assess the likelihood of multiple primary malignancies (MPMs). Studies have shown that MPMs are present in nearly 8% of cancer survivors (6), with the overall survival rates for dual lung–prostate primary cancers being significantly lower than that for single cancers (7), and that early and accurate diagnosis is essential for improving prognosis. In this paper, we report on a case of a 74-year-old male patient who was diagnosed with cervical lymph node metastasis of PCa, with respiratory symptoms as the first manifestation, and highly suspected of adenocarcinoma of lung origin by multimodal examination. The special features of this case were as follows: 1) as the first metastatic sign of PCa, the cervical lymph node involvement lacked the transitional stage of typical pelvic metastasis, and 2) the lung lesion presented a rapid response to pemetrexed chemotherapy, which is not in line with the conventional treatment response pattern of metastatic lesions of PCa. By systematically describing the diagnostic and therapeutic decision-making process of this case, this report aimed to provide a molecular diagnostic basis and a therapeutic strategy optimization idea for the clinical management of similar complex cases.

## Case presentation

**Figure 1:**
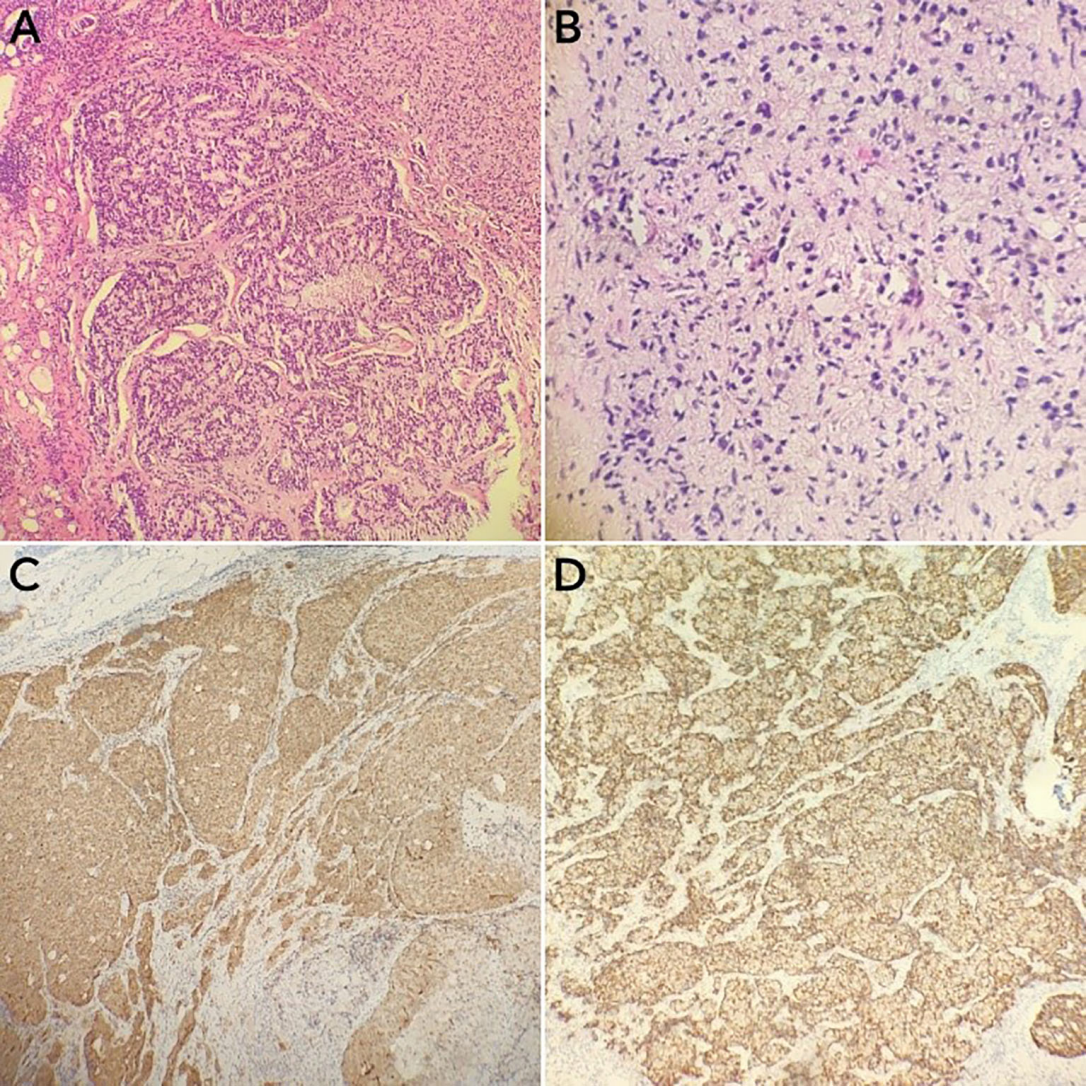
Immunohistochemical specimens of the cervical lymph node puncture pathology and the prostate puncture biopsy pathology of the cervical lymph node. **(A, B)** Hematoxylin–eosin staining of the cervical lymph node **(A)** and the prostate **(B)** puncture biopsy tissues (×100). **(C, D)** Prostate-specific antigen (PSA) **(C)** and P504S **(D)** immunostaining-positive tumor cells (×100).

**Figure 2:**
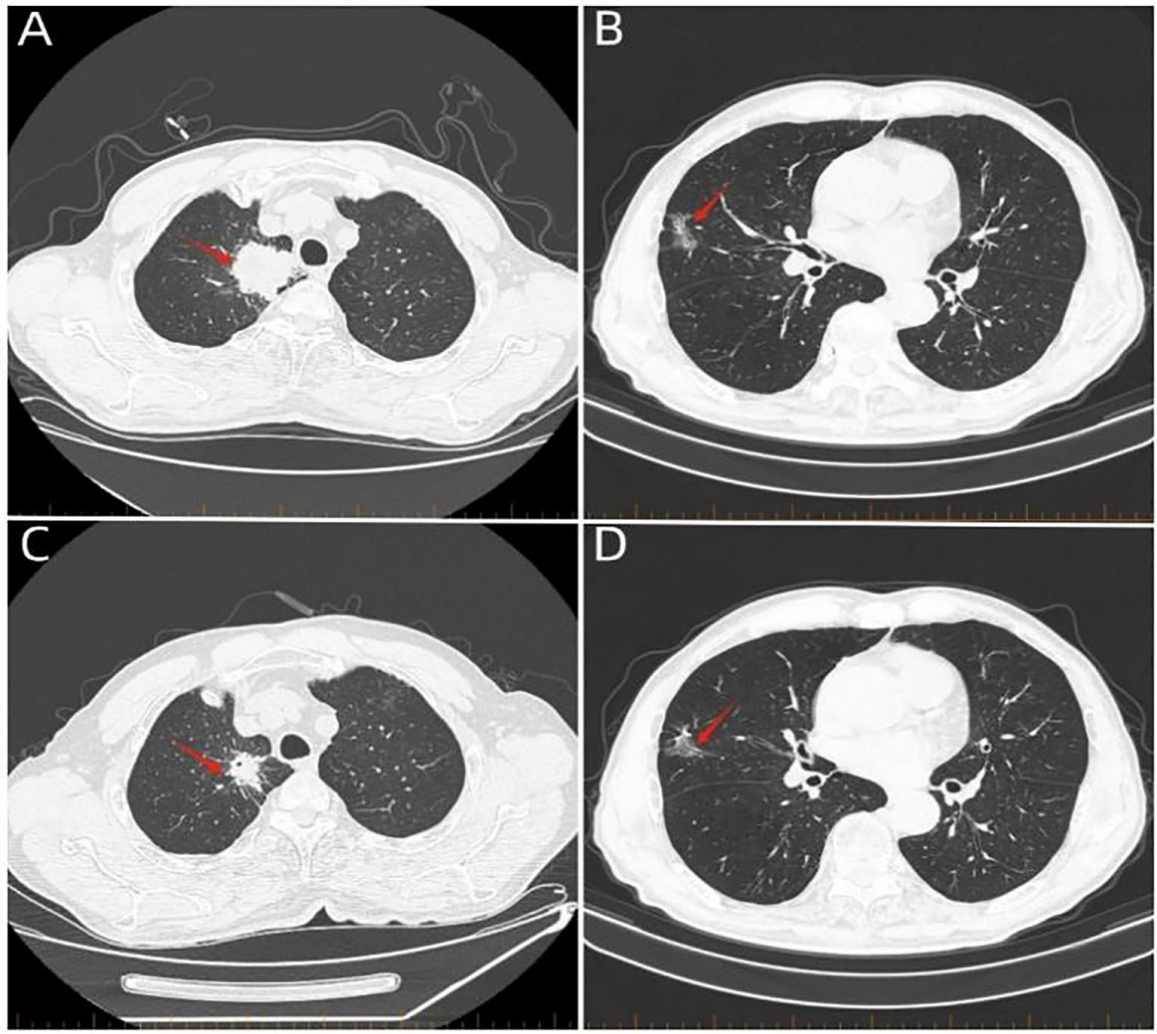
Whole-body computed tomography scan. **(A)** Computed tomography scan on March 16, 2023, showing a 6.4-cm × 4.7-cm mass in the apical segment of the right upper lobe of the lung (*red arrow*). **(B)** Computed tomography scan on March 16, 2023, showing a mass in the middle lobe of the right lung (*red arrow*). **(C)** Computed tomography scan on June 6, 2023, showing a 3.1-cm × 2.4-cm mass in the apical segment of the right upper lung (*red arrow*). **(D)** Computed tomography scan on June 6, 2023, showing the middle lobe of the right lung with an occupancy (*red arrow*). A 3.1-cm × 2.4-cm mass is seen in the apical segment (*red arrow*).

**Figure 3:**
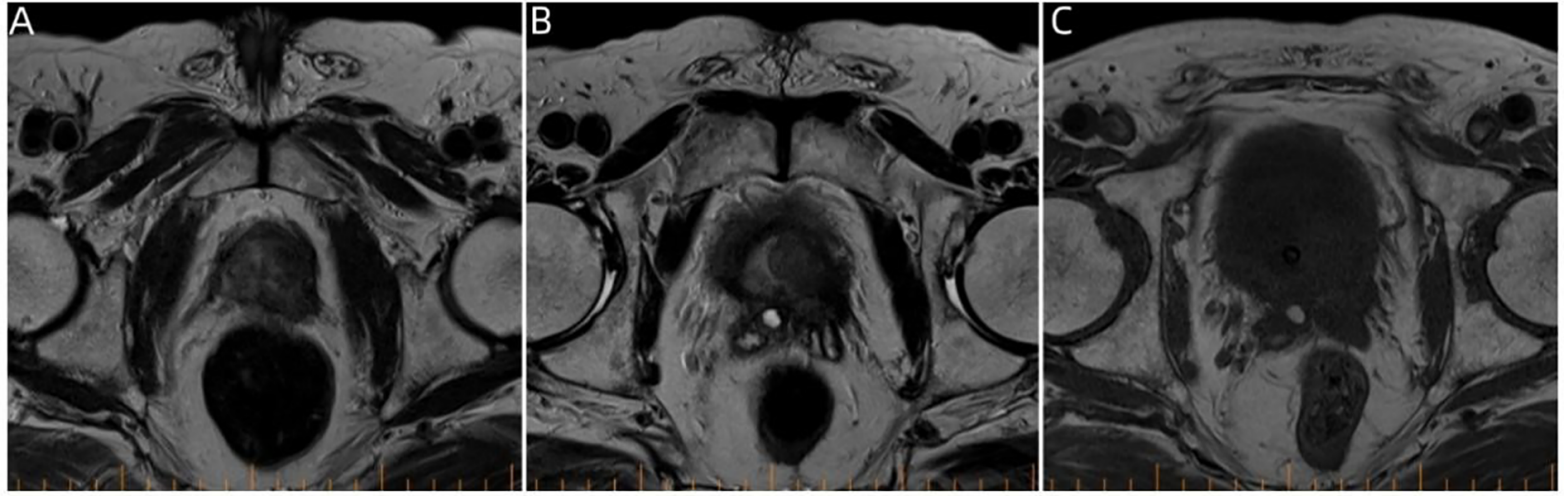
Pelvic magnetic resonance imaging. **(A–C)** Prostate magnetic resonance imaging findings on January 8, 2024, showing multiple round-like mass shadows within the prostate migratory zone thereof, with the central gland poorly demarcated from the peripheral zone.

**Figure 4:**
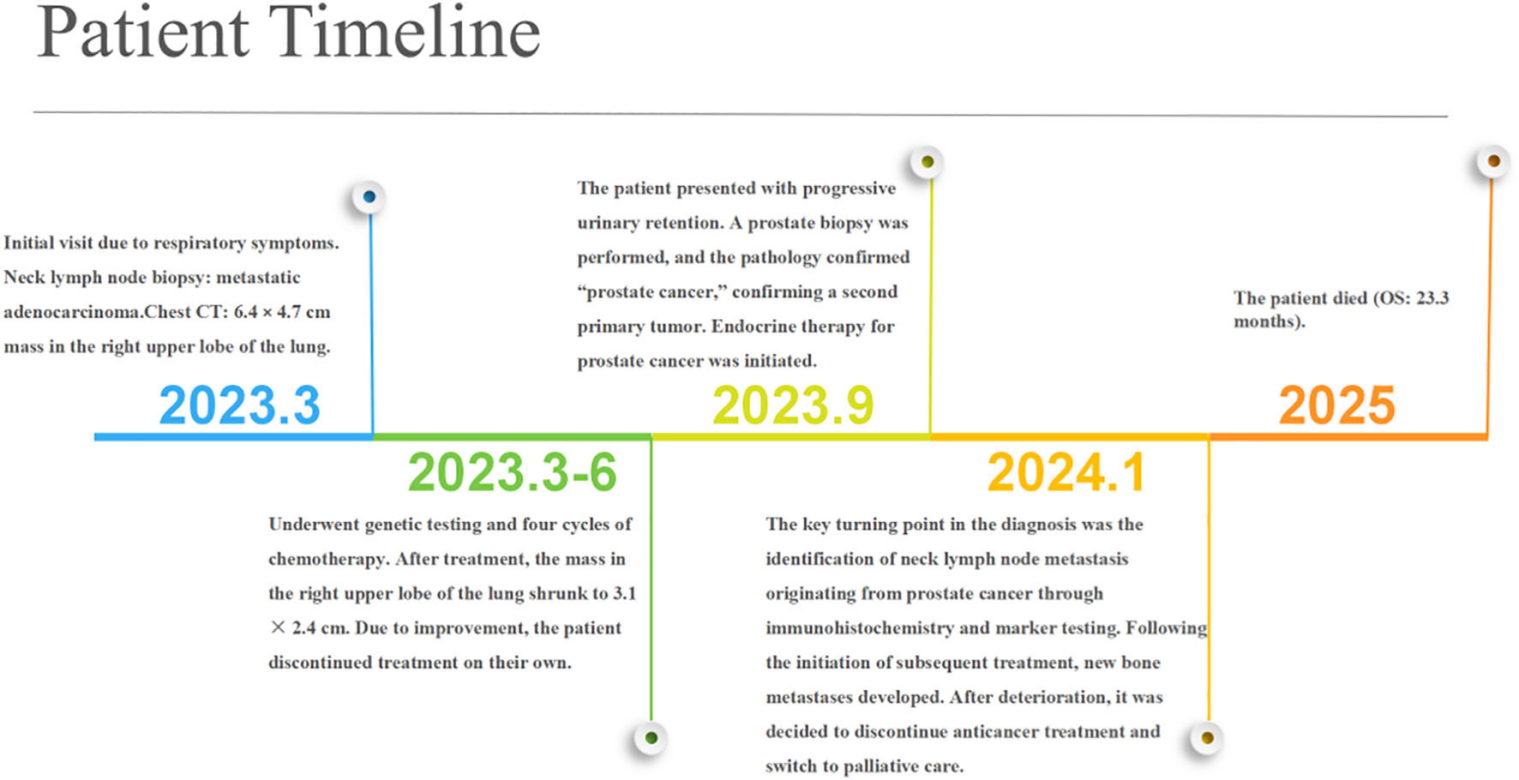
Disease timeline chart of the patient.

A 74-year-old male patient presented with cough and sputum with intermittent blood in the sputum without obvious triggers. He was seen in a local hospital, where a computed tomography (CT) examination showed a right upper lobe lung mass, which was left untreated. He was seen in the Department of Thoracic Surgery of Xinhua Hospital, affiliated with Dalian University, on March 15, 2023, where he was treated with a biopsy of the cervical lymph nodes. The pathology showed metastatic adenocarcinoma (**Figure 1A**), and genetic testing found no mutations in *EGFR*, *ALK*, *HER-2*, *ROS-1*, *RET*, *NTRK*, *BRAF*, and *MET*. The following day, a chest CT was performed, which showed occupancy in the upper lobe (**Figure 2A**) and the middle lobe (**Figure 2B**) of the right lung. In addition, a 6.4-cm × 4.7-cm mass was seen in the apical segment of the upper lobe of the right lung, within which speckled calcification was observed, with an irregular margin and short burrs. The enhanced scan showed an uneven and obvious enhancement of the lesion, which was closely related to the adjacent pleura with an unclear demarcation (**Figure 2A**), and occupancy in the middle lobe of the right lung. Chemotherapy with pemetrexed + carboplatin + bevacizumab was administered for four cycles. A follow-up chest CT on June 6, 2023, showed a 3.1-cm × 2.4-cm mass in the right upper apical segment of the lung (which was significantly reduced compared with that observed on March 15, 2023) (**Figure 2C**), and the lesion in the middle lobe of the right lung was roughly similar to the previous one (**Figure 2D**). The patient’s symptoms such as coughing, coughing up sputum, and coughing up blood were significantly relieved after the treatment, and he stopped the treatment on his own. In September 2023, the patient was admitted to our hospital due to progressive urinary difficulty, and the pathology was suggested: prostate cancer (**Figure 1B**). He was given bicalutamide and leuprolide for endocrine therapy. On January 8, 2024, prostate magnetic resonance imaging showed that the prostate gland was slightly enlarged, approximately 4.3 cm × 3.8 cm × 2.6 cm, and the prostate peritoneum was poorly illuminated. The prostate migratory zone showed multiple rounded masses, with the larger ones measuring approximately 1.8cm × 1.4 cm, presenting a low signal on T1-weighted imaging (T1WI) and a slightly high signal on T2-weighted imaging (T2WI), with a poorly demarcated central gland and peripheral zone. The peripheral zone was not clearly demarcated. The central gland and the peripheral band were not clearly demarcated. The peripheral band showed a mass shadow of approximately 1.6 cm in length at 5 o’clock, which broke through the peritoneum and had a slightly restricted diffusion. The border was not clear (**Figures 3A–C**). Pathology consultation of the prostate specimen was given, which was consistent with a diagnosis of PCa, supplemented with immunohistochemistry of the cervical lymph nodes, prostate-specific antigen (PSA) (part 3+) (**Figure 1C**), P504S (3+) (**Figure 1D**), TTF1 (−), CK7 (−), CK20 (−), napsin A (−), and P40 (−), consistent with metastatic carcinoma of the prostate. The patient was recommended to further undergo bronchoscopy or percutaneous lung puncture biopsy in order to clarify the pathologic diagnosis and treat the lung cancer according to the pathologic diagnosis. However, the patient had peripheral lung cancer, and the lung puncture biopsy had a high risk of pneumothorax due to the long puncture path. Moreover, the tumor tissue could not be retrieved by bronchoscopy. Therefore, the patient’s family members refused to accept lung puncture biopsy and bronchoscopy and gave up the further genetic testing and molecular targeting therapy. They also refused to accept tumor resection of the right upper lobe of the lung and demanded that chemotherapy and immunotherapy be selected for the chemotherapy regimen for the lung cancer. Considering that patients with PCa can survive for a long time but those with lung cancer have a short survival period, and due to the patient having developed a weaker breath sound in the right lung compared with the left lung, we chose docetaxel, which is an effective chemotherapeutic drug for both PCa and lung cancer, combined with the immunotherapy drug karelizumab. The patient started two cycles of docetaxel + karelizumab on January 10, 2024. The review after treatment showed that the lung lesions were reduced and the prostate lesions, the neck lymph nodes, and other metastatic lesions in the lung were all stable. As the lesion in the upper lobe of the right lung was considered to have a high probability of being a primary cancer of the lung, the treatment was therefore effective. All other lesions were considered to be metastases of PCa. It is worth noting that, in the course of chemotherapy, new bone metastatic lesions appeared, and the possibility of the bone metastases originating from PCa was considered to be high. The disease progression of the patient is shown in **Figure 4**.

## Discussion

This case presents three clinical topics that need to be explored in depth: 1) the atypical biological behavior of the cervical lymph node metastasis of PCa as the first metastatic sign; 2) the systematic path of differentiation when combined with the lung malignancy; and 3) the optimization of therapeutic strategies in the context of dual malignancies. This case provides an important reference for understanding the mechanism of atypical metastasis in urologic tumors and decision-making for the treatment of multiple primary cancers.

### Biological characteristics of atypical metastasis of prostate cancer

Lymph node metastasis of PCa generally follows a stepwise spread pattern, and the majority of cases first involve the occlusal–intra-iliac lymph node group (8). In this case, the first manifestation was the deep subcervical lymph node metastasis, and there was no anatomical evidence of pelvic lymph node involvement, which is consistent with “transregional metastasis.” Albadri and Salomão (5) suggested that this phenomenon might be related to abnormalities of the jugular–thoracic duct lymphoid reflux system. The CXCR4/CXCL12 axis may mediate lymphatic homing in PCa metastasis; however, its specific role in mediating cervical transregional metastasis requires further investigation (9). Notably, the metastatic foci of this patient showed TTF1 (−), P40 (−), and napsin A (−), excluding a lung cancer origin, but demonstrated strong P504S positivity (3+) and partial PSA positivity, confirming a prostate origin, suggesting tumor clonal heterogeneity, which may stem from the downregulation of the androgen receptor signaling pathway (10). This provides a molecular rationale for the subsequent use of docetaxel in combination with immunotherapy.

### Differential diagnostic challenges of combined lung occupancy

The lung lesion presented three paradoxical features: 1) the volume reduction rate after pemetrexed + carboplatin chemotherapy was >50%, which was significantly higher than the conventional response rate of metastatic prostate cancer, and pemetrexed and carboplatin are not therapeutic agents for PCa; 2) CT showed typical signs of malignancy (i.e., short burrs and pleural tugging); 3) there was no correlation between the serum PSA level and the regression of the lung lesion; and 4) after docetaxel treatment, the systemic lesion response was inconsistent, with partial regression of the lesion in the upper lobe of the right lung, but no regression of any other lesion. Although histologic confirmation of the diagnosis could not be obtained due to the patient’s refusal to biopsy, the chemotherapy sensitivity assessed according to the RECIST criteria, along with typical imaging findings on CT, was more supportive of the possibility of adenocarcinoma of lung origin. This diagnostic dilemma highlights the importance of liquid biopsy techniques, and circulating tumor cell (CTC) phenotyping can effectively differentiate between epithelial cells of lung/prostate origin (11). In future clinical practice, it is recommended that methylation profiling of the circulating tumor DNA be incorporated into the differential diagnosis process. In addition, immunohistochemical techniques should be fully utilized in pathological diagnosis to carefully determine the origin of the metastases.

### Systematic consideration of dual malignancy treatment decision-making

The “two-way coverage” strategy adopted by the treatment team is innovative: 1) docetaxel can target the microtubule system of PCa (12) and plays an antitumor role by inhibiting the Bcl-xL protein in lung cancer cells (13); 2) the application of karelizumab was based on the newly discovered aberrant expression of programmed death-ligand 1 (PD-L1) in peri-nervous infiltration (14); and 3) dynamic monitoring of PSA and carcinoembryonic antigen (CEA) dual indexes provides a multidimensional basis for efficacy assessment. Notably, despite partial remission of the lung lesions, the appearance of new bone metastases suggests the influence of tumor heterogeneity on the therapeutic efficacy. PET/MRI-based whole-body metabolic assessment may be helpful for the early detection of such “fugitive lesions.”

Although we designed a therapeutic strategy intended to target both tumors and observed a partial response in the thoracic lesions, the overall clinical course of the patient was ultimately dominated by the aggressive biological behavior of his PCa. The development of new bone metastases during combination treatment with docetaxel and karelizumab represents a stark manifestation of tumor heterogeneity, signifying dissociated response and therapeutic resistance. Following completion of the two treatment cycles, given the limited efficacy of further systemic antitumor therapy, the significant treatment burden, and the poor overall prognosis, the medical team engaged in thorough communication with the patient and his family. Ultimately, based on respect for the patient’s wishes, a joint decision was made to discontinue all antitumor treatments. Thereafter, the focus of the patient’s medical care shifted from antitumor-directed therapy to palliative care centered on symptom control and quality of life enhancement. The patient ultimately succumbed to his disease on February 21, 2025, with an overall survival of 23.3 months from initial diagnosis. This outcome underscores the profound clinical challenges posed by multiple primary malignant tumors and emphasizes the paramount importance of early and sustained integration of palliative care principles and shared decision-making models into the entire management process for patients with advanced refractory cancer.

## Conclusion

The clinical analysis of this case yields several key insights: when diagnosing lung space-occupying lesions in elderly male patients, it is imperative to move beyond the paradigm of “monism” and instead focus on a differential diagnosis approach, with a particular emphasis on the screening of tumor markers such as PSA. In instances where a histopathological diagnosis is not feasible, a cautious approach to therapeutic diagnostic strategies should be adopted, utilizing insights from both pharmacokinetic (PK) and pharmacodynamic (PD) characteristics to identify and treat the primary tumor foci. Furthermore, investigating targeted therapeutic options that span the tumor spectrum, such as antibody–drug conjugates (ADCs) targeting the TROP2 antigen, may offer innovative treatment modalities for the management of MPMs. Consequently, it is suggested that subsequent studies undertake whole-exome sequencing of PCa neck metastases to elucidate the potential role of the CXCR4/CXCL12 signaling axis in this atypical pattern of metastasis.

This case serves as a profound reminder that, when evaluating suspected secondary tumors, one must be cautious about drawing conclusions based solely on morphological similarities. Any preliminary morphological assessment must be rigorously validated through advanced diagnostic techniques (such as immunohistochemistry and molecular pathology, among others) in order to establish a definitive diagnosis.

## Data Availability

The original contributions presented in the study are included in the article/supplementary material. Further inquiries can be directed to the corresponding author.
